# Exploring Daily Activity Patterns on the Typical Day of Older Adults for Supporting Aging-in-Place in China’s Rural Environment

**DOI:** 10.3390/ijerph17228416

**Published:** 2020-11-13

**Authors:** Ziqi Zhang, Zhi Qiu

**Affiliations:** 1 School of Design, Shanghai Jiao Tong University, Shanghai 200000, China; 2 Institute of Architectural Design and Theoretical Research, Zhejiang University, Hangzhou 310000, China; qiuzhizju@sina.com

**Keywords:** aging-in-place, older adults, daily activity patterns, rural environment

## Abstract

Severe aging in rural China is prompting communities to promote support for older people to age in place. The study of the daily life of older adults in rural areas is conducive to understanding their real life and demands, as well as the way they interact with their environment, to develop feasible strategies. In this study, 171 older adults over 60 years old in two different types of villages in Northern Zhejiang Province were investigated and analyzed in terms of the temporal and spatial features of daily activities, as well as their relationship with population attributes, personal competence, and subjective demands. The results show that: (1) significant association can be seen between working hours and the demand for health services, housework hours and gender and age, as well as leisure hours and ADL and the demand for recreational services. (2) The older adults appear to have inter-group homogeneity in some aspects: basic living activities, leisure hours, the gender difference in housework hours, and recreational preference, while they have higher average paid work hours and fewer leisure alternatives than their urban counterparts. Their definitions of paid work, housework, and leisure activities are vague. (3) The definition of home by the older adults in rural places sometimes seems to go beyond the scope of their own house, and the extensive definition of home may change their recognitions of some activities. They also inclined to assign meaning to a place through frequent use rather than through external definitions. (4) The weak consciousness on buying services and deteriorated financial situation hinders the older adults in rural communities from expressing their real demands. Unspoken demands include economic security, recreational choices, and assistance in housework. The results will help to provide references for the improvement of eldercare services and the community environment.

## 1. Introduction

With China entering the super-aging period [1], it is estimated that the number of aging people in China will reach 418 million by 2035, accounting for 39% of the world’s older population [2]. Rural China is facing a more severe situation, where the aging rate has become even 5% higher than in urban areas [3,4]. Filial piety, intergenerational ties, and family cohesiveness, which have long been rooted in Chinese traditional culture, strongly influence the older adults’ preference for aging-in-place (AIP) [5,6,7]. AIP refers to the possibility to live in one’s present non-healthcare environment till life ends by accessing multiple products or services within communities [8]. In order to cope with this situation, China has implemented the 9073 or 9064 model since 2009 to reach 90% of the older adults aging in their local communities [9]. However, in rural communities, shrinking family size and the out-migration of young people have strongly destroyed the informal social capital for caring for the aging adults [10,11,12]. Meanwhile, successful aging-in-place is also hindered by the lower-quality eldercare infrastructure that mismatches with the rural elders’ characteristics and needs [13,14,15,16].

The aging process is usually explained from the perspective of an interaction between the individual and the environment [17,18]. Under the influence of humanism, the research from a micro perspective on daily activities and social space is becoming a significant perspective for interpreting people and place [19]. Reliable data describing the daily life of older people, such as content, location, and the company of activities, can provide the basis for a better understanding of the aging process and thus contribute to successful aging [20,21]. The measure of spatiotemporal features also allows for better estimation of service demand [22]. Due to physical limitations and socioeconomic disadvantages, the daily activity patterns of older people are not consistent with those of other age groups [23]. Previous literature demonstrates a mounting interest in the characteristics of travel behavior, mobility, space–time budget, and life space of the aged [24,25,26]. Some Chinese research has shed light on the temporal and spatial patterns of medical, shopping, or leisure activities of older adults in Shanghai, Beijing, Nanjing, and other metropolitan areas [23,27,28]. Large-scale data sets on the elder’s time use are also being developed in Canada, Australia, the United Kingdom, and some Asian countries [29,30,31,32], identifying national differences in time use patterns of the elder population. Studies on China have studied the time use allocation of agricultural and non-agricultural, paid work, and housework of the senior group [33,34].

These results are valuable in understanding the characteristics of older adults’ daily activities [35]. However, on the one hand, although rural areas are considered to be of greater significance in understanding the aging process and outcomes [36,37], relevant discussions focus on cities [38,39,40,41]. On the other hand, while large-scale statistical modeling allows for trend mapping, programmed questionnaires, which serve as the main approach to capture time use data in this research, might provide normatively but unprecise answers compared to diaries [42,43,44]. At the same time, these studies usually record only primary activities, ignoring simultaneous or so-called secondary activities, which are essential for accurate estimation of time use [45,46]. Since universal features are accumulated from individual activities [47], studies of older adults’ daily lives from a micro-location perspective can contribute to an in-depth understanding of the complexity and dynamics of life experiences, tensions, strategies, and expectations of those experiencing these changes [48,49,50,51]. The challenge is not to over-represent or under-represent the needs of rural older people from myths and stereotypes, but to perceive their real life in an unobtrusive way [41,52,53].

Therefore, the purpose of this study is threefold: (1) to explore the daily activity patterns of the “typical day” aggregated by the individual experience of rural older adults; (2) to analyze the relationship between different types of daily activity patterns and individual factors, including population attributes, personal competence, and subjective demands; (3) to discuss the characteristics and demands of rural older adults reflected in their daily activities in order to provide references for aging-in-place strategies in rural contexts.

## 2. Research Methods and Areas

### 2.1. Theoretical Framework

Basic human desires, including earning a living, socializing, or having fun, motivate different types of activities. The core concept of the needs–satisfaction model is that each activity of the personal repertoire aims to meet specific demands [54]. Previous literature has focused on the short-term dynamics of the demand and activity generation process [54], heterogeneity in activity participation among older adults through the difference in perceived satisfaction over a given time period (for example, an average day of a person) [55], and the impact of demand satisfaction on the decision-making of time use [55,56,57]. Lawton’s ecological theory [17] elucidates the significance of the interaction between environmental press and personal competence for successful aging. Individual-level characteristics or group heterogeneity will also affect or restrict participation in activities and time allocation [47,55,58,59,60,61,62]. Moreover, people will weigh the time allocated to different activities within a time limit [63,64]. More important and sticky temporal fixation activities will bind a series of behaviors to a specific place and time, limiting an individual’s opportunity to engage in a variety of activities [65]. Separate studies of certain types of activities (e.g., work, leisure, or shopping) may ignore the intrinsic influence of activities on time allocation decisions [55].

In addition to the mutual constraints of demands, personal competence, and attributes, the studies in environmental gerontology also explicitly consider how the environment affects the aging experience, and partially stimulates the study of describing, explaining, modifying, or optimizing the relationship between aging people and their physical environment [66]. The concept of “place” in aging-in-place is constantly under examination and retrospection from a narrow sense of residential area to a mesoscale of “life-space” [67,68], which is essential to examine the interrelationship between aging, place, and health. It can also be defined as a neighborhood [69,70], or a “home beyond the house” that generates belonging, identity, and place attachment in the relationship between people and the environment [18,71]. At the same time, the view of the transaction has changed from perceiving “place” as the container of human activities towards understanding that place and human are shaping together and inseparable from each other [69,72,73,74], and how older adults transform meaningless geographical spaces into places with personal significance and identity [75].

On this basis, this study considered the possible impact of population attributes, personal competence, subjective demands, and time constraints from other activities on daily activities. At the same time, this study also examined the locations of activities and how older adults transform places into a part of their living space through transactions.

### 2.2. Research Methods

**Figure 1:**
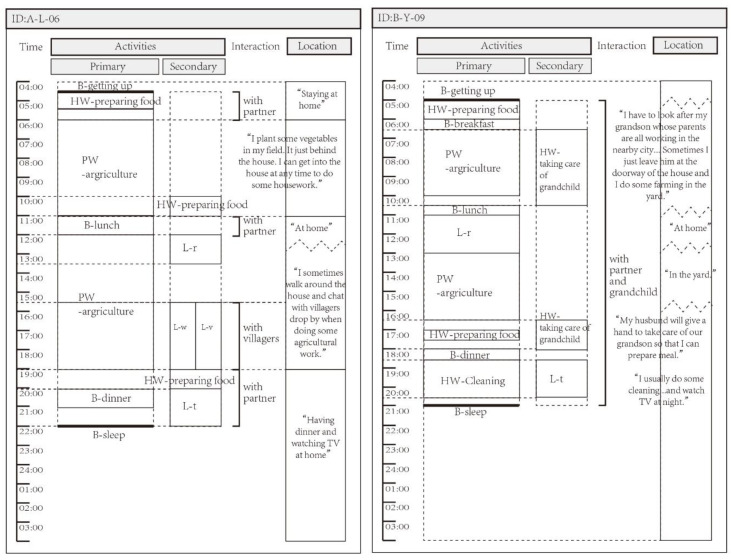
Prepared forms and on-site recordings of the activities and locations.

According to the research framework, the investigation consisted of the following:

(1) A population attributes inquiry including age, gender, education, marriage, religious belief, and living arrangement. In this study, an older adult in the rural environment was defined as a rural permanent resident aged 60 years or older.
(2) The ability assessment of the aged issued by the Ministry of Civil Affairs of China for evaluating personal competence. The activities of daily living (ADL), mental state, perception, communication, and social participation of the older adults were assessed, respectively, and the results were categorized into independent and with disability.
(3) A structured questionnaire for accessing the degree of subjective demand from five aspects of daily care, medical care, education, recreation, and self-presenting services represented by 21 sub-items and a five-level Likert assessment (reliability = 0.743 > 0.7). For better calculation, the result was re-categorized into two levels: in need or indifferent, and not in need.
(4) Prepared forms for recoding daily activities of the “typical day”. The systematic recording of an individual’s activities, including the sequence, time point, duration, etc. in a specific short period of time (such as a 24-h cycle) is called the time budget or patterns of daily occupations [76]. The time–space budget was developed on the basis of the time budget, which contains the spatial information related to the location of activities [77,78]. Data for this research was mainly collected through an open-ended or pre-arranged activity diary, and participants were required to report what they were doing over a certain period of time. The extended activity diary also recorded data on locations, relationships, and other expectations of daily occupations [79]. Using the method of a recall diary, i.e., we asked the respondents to recall the activities on their “typical day” and reconfirm whether they think the recorded date was “the most common” to exclude the “special” date from the analysis [44]. Although post-event recall may be biased due to memory limitations of older adults, the false reconstruction [80] can instead be considered as an “aggregation” of longer-term daily experience. This method is helpful to acquire more reliable, non-interference, and as many sample data as possible. In terms of coding for activities, the classic time allocation model divided activities into two categories: work and non-work [63], and there were also occupations engaged in maintenance, work, rest, or leisure [44,81]. According to the pre-survey, we coded the activities as below: basic living (B), paid work (PW), housework (HW), and leisure activities (L). Leisure activities include watching TV (L-t), card games (L-c), dancing and music (L-m), taking a walk (L-w), taking a rest (L-r), cultural activities (L-a; reading, radio, internet, etc.), and chatting (L-v). The main activity and secondary activity, as well as descriptions of the activities, were also recorded (Figure 1).

According to the data from the Zhejiang Provincial Bureau of Statistics, the population aged 60 years and above is 11.244 million, accounting for 19.6% of the total population, and has entered a deep aging society [82]. The urgency of the problem and the overall economic level (a GDP of 5619.7 billion yuan in 2017 [83]) make Northern Zhejiang of practical significance to take this area as the research context. Considering that the daily activities of older adults may be affected by the economic development level or the dominant industry, two rural samples with different levels of development were selected to reduce bias in the conclusion. Among them, village A is located in Anji County in the north of Zhejiang Province, with an area of 1.562 square kilometers and a permanent population of 1894. It is a typical traditional agricultural village with the primary industry accounting for 94.2%; village B is located in Deqing County in the north of Zhejiang Province, with an area of about 6.8 square kilometers and a total population of 1395. It is a village with tourism and other tertiary industry as the main economic source. According to the 2018 Huzhou GDP data, Deqing County and Anji County represent different economic levels.

This study was part of a larger research project on the aging situation and corresponding strategies in rural areas of the Yangtze River Delta conducted from April to July 2017. In this study, we first conducted a pre-survey in 20 villages along the coast of China, including observations and surveys (5–10 records per village) of the older villagers (age ≥60), as well as open interviews with those who were in charge of senior affairs. In order to minimize the possible impact of weather and other external environments on the results, the main part of this study (daily activity patterns on the typical day) was completed within one day (22 June 2017).

Twenty students were trained in the administration of the questionnaire by the researchers. As most of the rural older adults have limited educational levels, the 1~4 investigations could only be conducted by one-to-one interviews. The narrative interview was based on the open narrative heuristic process and the guiding principles. Questions like “when do you get up and have meals in most cases?”; “what do you usually do in the morning?”; and “do you take a nap at noon? How long do you sleep?” were used to construct the most common (“typical”) day of the respondents. In order to reduce the interruption of recording time on the respondents’ thinking, the interview was quickly marked by coding and fully recorded. Raw materials were proofread and analyzed by the two authors through group discussions afterward, and we optimized the data collection and analysis through collective reflection [84].

### 2.3. Statistical Analysis

In this study, descriptive statistics were used to reveal the characteristics of daily activities of the rural elderly. All parameters were transformed into categorical variables, and the chi-square test was conducted to examine the possible association between the characteristics of activities (core variables) and the characteristics of a person (sample background). SPSS 17.0 for windows was used for statistical analysis, and a *p*-value < 0.05 was considered to be statistically significant. K-means cluster analysis was also used to categorize the aging groups according to daily activity patterns. For the qualitative study, the first step was to classify by the keywords of location (such as explicitly referring to home or a public facility). An advanced analysis was used to discriminate some ambiguous locations in sentences, and to redefine and classify these words through on-the-spot records of the respondents (for example, when referring to words such as “over there“ or ”nearby”, the respondents were asked to identify the specific place on the map).

### 2.4. Investigation Summary

Restricted by reality condition, 200 eligible participants were approached by convenient sampling, among which 29 participants had missing information or were unwilling to answer certain questions, leaving 171 subjects available for the present study. The basic research was as follows (Table 1).

## 3. Results

### 3.1. Temporal Characteristics of the Daily Activity of Older Adults in Rural Areas

**Figure 2:**
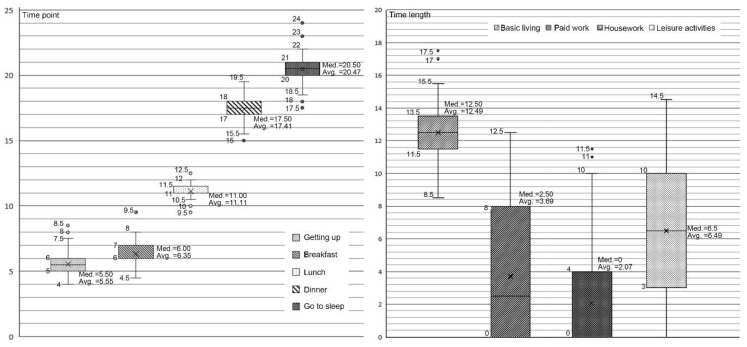
Temporal characteristics of daily activities of older adults in rural areas.

The temporal features of rural older adults’ basic life patterns are shown in Figure 2: (1) The average time of getting up was about 5:30, with 50% of the older respondents getting up at 5:00–6:00 and going to sleep at 20:00–21:00. The length of sleep at night was a basically normal distribution, with the maximum being 8.5–9 h. Breakfast, lunch, and dinner were usually taken from 6:00–7:00, 11:00–11:30, and 17:00–18:00, respectively; (2) In terms of paid work, 57.3% of the respondents were still working, with the length ranging from 0 to 12.5 h a day, and the overall average was 3.69 h. Among the working group (52%), 50% of them worked 4.25 to 9.25 h, with an average of 7.1 h per day. The main jobs were agriculture (68.3%), being employed (15%), and village- or religious-related affairs (9%); (3) In terms of housework, 45% of the respondents reported housework time, ranging from 0 to 12.5 h, with an average of 2.07 h. The average time spent on housework was 4.6 h, and 50% of the respondents worked between 2 to 7 h. Household chores reported included laundry and cleaning, shopping and preparing meals, and taking care of others (e.g., raising grandchildren); (4) 94.7% of the respondents reported leisure hours, with the upper and lower limits of 1 and 14.5 h and an average of 5.49 h. The most common leisure alternative was watching TV (reported by 64.9% of the respondents), followed by resting at home (49.1%).

The working hours could be further divided into four categories: zero hours, less than four hours, four to eight hours, and more than eight hours (Table 2). The older adults in village A had a higher proportion in both nonworking and long-time working categories. The observed values of men, younger (60–69), and single old people who did not work were less than the expected values, and the adjusted standardized residuals were −1.5, −2.4, and −2.2, respectively, indicating that such people were not inclined to be nonworking. There was a significant correlation between the demand for health service and the length of working hours (*p* = 0.006). The demand for health service indicated the respondents’ evaluations of their own health. People who thought they needed health services did not tend to work for a long time (>8 h).

Similarly, housework hours and leisure hours were divided into several categories. The chi-square test showed that the housework hour was significantly related to gender (*p* = 0.042) and age (*p* = 0.002). The adjusted standardized residuals were 2.4 for women and 1.6 for men, indicating significant gender differences in housework hours. By adding the hierarchical chi-square of the location to test the relationship between gender and housework duration, we found that the gender significance of housework only appeared in the traditional agricultural village. In terms of age, the older adults in the middle age (70–79 years old) tended to do housework for a long time (>4 h), while the younger older adults (under 70 years old) tended not to do housework.

For the daily duration of leisure activity, men, the oldest of the old, alone or widowed, with a mental state and perception communication disabilities, and those with adequate social participation ability tended to have longer leisure hours, and those who had no demand in basic care, education, or self-fulfillment were just the opposite. Leisure time was significantly correlated with the ADL situation (*p* = 0.047) and the demand for recreational service (*p* = 0.043). The proportion of people with longer leisure hours was higher in the ADL disability group than in the independent group.

### 3.2. Spatial Features of Daily Activities of Older Adults in Rural Areas

**Figure 3:**
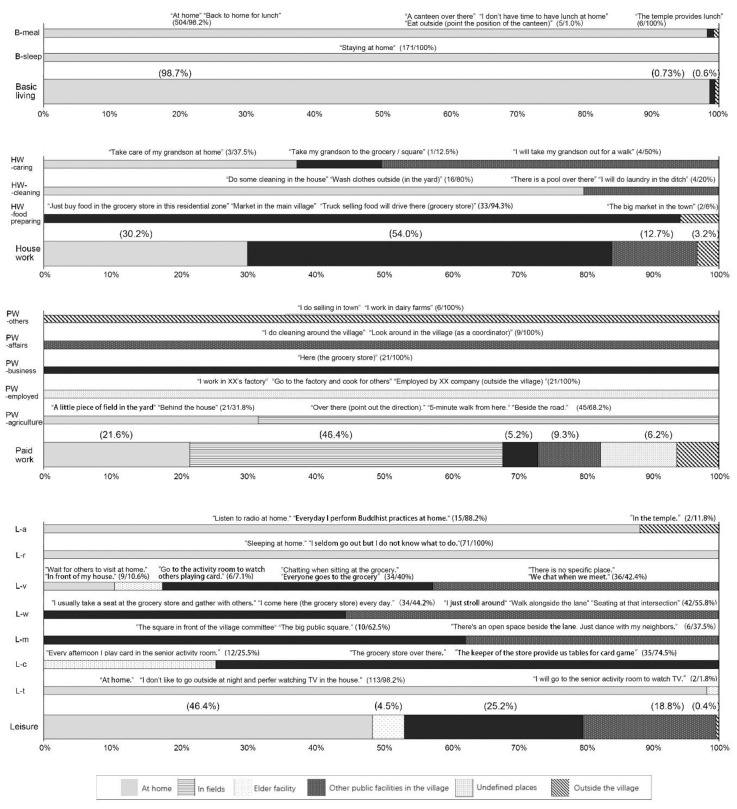
Sample words and categories representing the locations of daily activities.

The description of where the activity took place may reflect the concept and attitude of older adults towards the location. Words representing the location of the activity were recorded and categorized (Figure 3). Most of the basic living activities took place at home and could be hardly found in daycare centers or senior homes, especially in Village B. Many respondents said that only “abandoned people” would have to live outside of the home. On the other hand, the respondent B-L-11 expressed dissatisfaction with the service quality of the existing eldercare facilities in the village. The respondent B-L-04 held that at present, “the senior homes can only serve healthy older adults due to the lack of substantive care, which makes these facilities meaningless for me”. There was a small canteen set up by the villagers in Village B that could benefit the elder workers. The 71-year-old respondent A-W-11, who worked for up to 10.5 h per day from Village A, proposed, “a public canteen at a reasonable price would be good for reducing the burden of housework at least”.

Most of the agricultural work took place in their own fields, and the locations included fields within the scope of “home” (such as the backyard or adjacent places of housing) and fields far away. The location of the fields may also affect the activities of older adults in positioning agricultural work. The agricultural work taking place “at home” was more likely to be described as a kind of “leisure” for killing time, and also more likely to take place with secondary activities, such as B-L-06 said, “there’s nothing to do during the day. I plant vegetables in my backyard and do housework as a pastime”. Taking labor jobs as a kind of leisure also occurred in other types of work, such as respondent A-Y-07, who was engaged in cleaner jobs in the village, said, “there are no other choices for leisure, so only work can make me keep healthy”. However, it is undeniable that self-support was still the reason why most older workers continued to work. For example, the respondent B-L-01 mentioned, “I find scattered land to plant some crops for my own use, and I will also buy some in the market. I just work and eat on my own and take care of myself”. According to the larger investigation, 31% of the respondents still had no pension security, and their livelihood had to depend on their children or themselves. Sometimes, they even needed to afford the living of their older parents. For example, A-L-07 still needed to work to earn money to care for the elder mother, who was paralyzed by a stroke.

Most elder respondents could basically achieve self-sufficiency of vegetables. There was a small market in village A, which provided limited alternatives to vegetables and meat. Some older adults rode battery cars to shop in the nearby town. The mobile selling vehicle arrived at about 9:00 every morning at the entrance of the grocery store, another approach to supply daily necessities. Four cases of cleaning (laundry) sites were reported in public ponds or river channels in the village. Taking care of grandchildren usually took place as a secondary activity with leisure or housework activities, and was likely to happen in public facilities, like grocery stores and undefined places (roadside, empty spaces, or road intersections).

### 3.3. Daily Activity Patterns of Older Adults in Rural Areas

**Figure 4:**
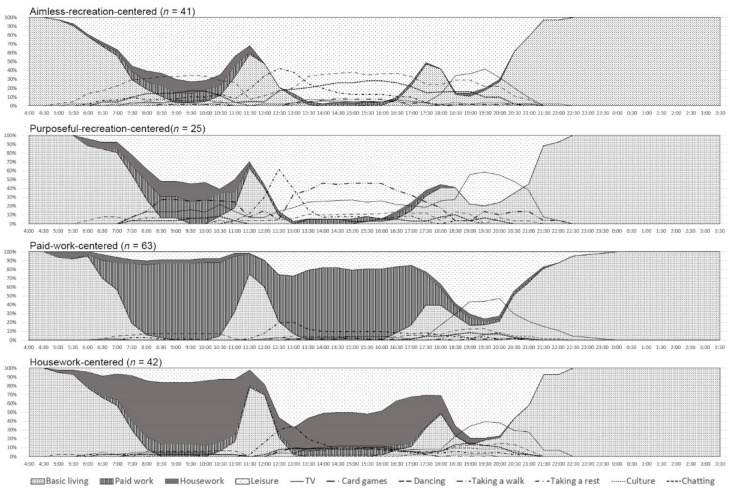
Four patterns according to daily activities (the numerical value of each time point is the proportion of people doing a certain activity in this group).

In order to consider the impact of time constraints, the older adults were clustered by working hours, housework hours, and seven kinds of recreational activity hours. After comparison, the number of clusters was four after seven iterations. According to the final clustering center, daily activity patterns could be divided into aimless recreation centered (*n* = 41), purposeful recreation centered (*n* = 25), paid work centered (*n* = 63), and housework centered (*n* = 42). The characteristics of the four types of people are shown in Figure 4. The numerical value of each time point is the proportion of people doing certain activities in this group.

As further studies show, the members in the aimless recreation centered group were more likely to fall into the category of Village B, man, older aged, nonreligious, low demand, and high ADL damage rate. This group also showed the highest single rate among the four groups. The biggest difference between the purposeful recreation centered group compared to the aimless recreation centered group was the lower single/living alone rate and the higher demands in all kinds of services. The paid work centered population was more likely to be in Village A. They were mainly men, with higher education levels, the lowest single rate, obtained an average of abilities, and had a higher demand for health and recreation. In addition, the majority of the housework centered group were female, younger age, maintained poor communication ability, and had a higher demand for health.

## 4. Discussion

### 4.1. Similarities and Differences of Daily Activity Patterns in Older Adults in Rural Areas

According to the results, the average paid working hours of the rural older adults (6.4 h) is far higher than that of the urban older adults (26 min [85]). Due to low-level pension security and the weakening of family support in rural areas, working is for survival. In addition, the trend that paid work and housework hours do not change with health status might indicate that the older adults have little awareness of their own physical condition, which may lead to a lagging demand for healthcare and disease prevention.

Time use patterns of rural households in developing countries reveal a significant division of labor based on gender and age [20,34]. The results confirm that women continue to spend more time on household chores than men well into old age [85]. To make matters worse, care responsibilities, not only for their own but also for their parents and their grandchildren, are borne by older female villagers. It should be noted that random errors and recall bias may be present in the results due to the identification of household chores and fragmentation of time [86]. In particular, when care takes place in the private sphere (within the family), it is usually perceived as obligation and emotional commitment, but not housework [87]. In this study, respondent A-Y-02 mentioned that he had to buy breakfast for his grandson in a nearby town. A-L-07 has to take care of his mother with a stroke and paralysis. A-Y-12 and A-D-07 were taking care of their grandson when being interviewed. However, none of them reported corresponding housework hours.

The daily leisure time of the older adults in Chinese cities is generally about six hours, with watching TV and walking the main content [88,89], and 6.5 h for older adults in European countries, with the most common leisure activities being sedentary (watching TV or listening to the radio) and contacting with relatives and friends [90]. This study shows that the average leisure hours per day of the rural older adults is also 6.49 h, which mainly include low-threshold activities, such as watching TV and taking a walk. Similar conclusions with other aging groups are the difference of time allocation and interests between genders [20,91,92,93,94]. Just like urban older adults [95], health status affects the preference of leisure activities of rural older adults. However, different from the conclusion that adverse changes in health will affect the frequency and the ability of leisure participation [94,96], this study shows that people with mild ADL disabilities have longer leisure time due to being “less engaged in work” and “in fair health condition”. In addition, although there is no significant association, the leisure time of the single older adults is longer than that of the married group. This is different from the conclusion that urban older couples have the longest leisure time and more positive behavior [89]. In general, unlike the urban and western society, the quality and choices of recreational activities for the rural older adults are limited. More than one respondent mentioned that they “have nothing to do”. It should be noted that the peculiarities and complexities of rural areas are also reflected in the definition and calculation of leisure activities. Many activities that are generally defined as paid work or housework, such as farming, village affairs, caring for grandchildren together, washing vegetables, and washing clothes in public ponds or roadsides, etc., are also regarded as opportunities to have leisure and social interaction. The problem that the same activity can be defined from different perspectives makes it difficult to calculate, which may also lead to the omitting or underestimating of some ambiguous activities.

### 4.2. Home, Neighborhood, and “The Third Place”

Home is undoubtedly the most important place in the daily life of older adults [97]. The definition of home for older adults seems to be sometimes beyond the scope of the house itself, as their own courtyard and even the adjacent fields may also be defined as home. “Home beyond the house” is a conceptual approach to show how older people can expand the dynamic areas between the family and the community by connecting with the community and the neighborhood [71]. The extension of this definition is found in this study to change the classification and definition of some activities of older adults.

The results also show that older adults generally do not choose public places outside their residential zones for non-purpose leisure activities (chatting, etc.). However, their purposeful leisure activities, such as card games and dancing, do not necessarily take place in the most convenient places. The choices seem to be more dependent on familiarity and social networks. They sometimes also avoid using spaces designed specifically for them [98,99]. This is a reminder that older people may value public space very differently than planners expect. Under the guidance of national policies, many “specific facilities for the senior group” have been built in rural China, but these facilities do not seem to be fully integrated into the real life of the elder villagers. In the newly built daycare center for the older adults in Village B, although the facilities were “appropriately” equipped, there were no reports about leisure activities. They are more likely to gather in public spaces that they are familiar with, with a sense of belonging and inclusiveness, which in this study refers to the local grocery stores in each residential zone, as also revealed in previous studies [69]. This reflects older people’s preference for self-selecting spaces, i.e., giving a place meaning through frequent use rather than through external definition [99]. On the other hand, this study also shows the importance of “transitory zones” [69], which are not destinations but inclusive public spaces where older adults feel a sense of belonging, and provide opportunities for direct and indirect contact with the community and its residents. The opportunities for communication are not prearranged, but inspired or supported by the built environment with certain characteristics, including street edge, intersection, the entrance of the residence, besides the water channel and ponds, etc. Through continuous transacting with the environment, the older adults can form a dynamic boundary between home and neighborhood, and transform the rural public space into “the third place” [100], constantly enriching the connotation of the place for aging-in-place [101].

### 4.3. Unspoken Demands

The investigation on the demand for services demonstrates that rural older adults generally have lower subjective demands. It is found that the older villagers are generally sensitive to economic expenditure and less receptive to purchase services. During the investigation, they often said, “is this service free?”, “I don’t have money”, and “only services that do not cost money are welcome”, showing strong worries about the cost. Some respondents find it more difficult to seek and receive help, just as previous research shows that women consider receiving help as a loss of independence and an invasion of privacy [102]. All in all, the lack of awareness and insufficient purchasing ability prevents the rural older adults from expressing their demands through a “survey”.

Fortunately, the research on daily activity patterns from observation and records could somehow compensate for this knowledge. First of all, the working hours of rural elders after old age show a strong demand for economic security. At present, pension security for rural older adults in China is still at a low level. Continuing to work is not an alternative but a necessity to subsidize their living expenses. However, long-term work may lead to the aggravation of health problems, while economic constraints make them refuse timely medical care, let alone disease prevention. Although the new rural cooperative medical system helps to reduce the health inequality of the adults in rural areas, it did not lead to a better self-evaluation of the overall health of older adults. There is also no evidence that the new rural cooperative medical system has reduced their out-of-pocket costs [103]. Once they cannot tolerate the disease, they are trapped in a vicious circle of pain and poverty.

Secondly, the high ratio of “resting at home” in leisure activities reflects, to some extent, the lack of leisure activities for older adults in rural areas. Many respondents felt that they did not know what to do except staying at home. Even some old people take work as a means of killing time when they do not have enough leisure options. It may be due to the lack of awareness of more leisure options and the limitations of the rural environment [90,104,105]. At the same time, the elder female may have to bear a lot of domestic work while earning wages. Therefore, external support should be provided for older adults to ease their burden of household chores and to alleviate the conflict between housework and work.

### 4.4. Implications for Eldercare Support in Rural Environment

The findings may provide references for the provision of eldercare services. For example, a different care service supply process according to the activity patterns the different elderly groups can be adopted to develop and optimize the working time allocation of service providers. At the same time, programming of both space and activities should be rationalized to avoid the mutual influence caused by different life patterns. Low-cost centralized meal services such as “senior canteens” should also be provided to release the burden of the housework of rural older adults.

Moreover, the knowledge gained from this study can help planners to recognize the sensitivity of place in promoting an aging-in-place community. As some researchers have noted, it may be age-friendly even though the built environment is not as “satisfying” as the planners think [106]. We should not simply assume certain purposes and meanings, but actively involve the older adults in the early stage of policy-making [98]. Therefore, older people’s demands should be taken into account and efforts should be made to maintain emotional connections, continuity, and a sense of belonging with the neighborhood environment that supports their well-being [107]. It also seems to be a more feasible strategy to find and enhance the spatial characteristics of the existing “third place” and “transitionary zones” of the village than to add more new facilities.

## 5. Conclusions

This research captures the situation of the daily life of older adults living in villages, reveals the contents and characteristics of basic living activities, paid work, housework, and leisure activities, as well as the relationship between the features of daily activities and their population attributes, personal competence, and subjective demands. The older adults appear to have inter-group homogeneity in some aspects, such as basic living activities, leisure hours, the gender difference in housework hours, and recreational preference, while they have higher average paid work hours and fewer leisure alternatives than their urban counterparts. Their definitions of paid work, housework, and leisure activities are vague. A significant association can be seen between working hours and the demand for medical services, housework hours, gender, and age, as well as leisure hours and ADL and the demand for recreational services. Underlying demands of rural older adults reflected in their daily activities include economic security, recreational choices, and assistance in housework. Moreover, the definition of home by the older adults in rural places sometimes seems to go beyond the scope of their own house, and the extensive definition of home may change their recognitions of some activities. They also inclined to assign meaning to a place through frequent use rather than through external definitions. The result may serve as references for further strategies supporting aging-in-place in rural communities and might help develop a global solution for the increasing Chinese elder immigrants [108]. The limitations of this study are as follows: (1) Although some methods are adopted to ensure “typical”, there is still no guarantee that the older adults are vulnerable to the influence of their recent daily arrangement when filling the recall diary; (2) “Non-typical” activities or trivial activities, such as participating in some festival events, going to the hospital, or visiting relatives in the cities, need to be supplemented by other studies; (3) Although in the process of recording, we noticed and tried to classify work, housework, and leisure activities by the attitude of older adults through mood and description methods, and it is likely to lead to errors since, as mentioned by Bimbi [109], it is difficult to grasp the subjective dimension of time, that is, the meaning given by objects to activities; (4) As non-statistical big data, this study has unavoidable limitations in collecting the data and in the universality of conclusions due to the broad definition of the village itself and the diversity of the older adults. Therefore, the understanding of the stratification and personalization of the elder population in China need to be enhanced in future research. The corresponding research in other regions needs to be carried out continuously to increase the sample size of such research and continuously verify the conclusions.

## Figures and Tables

**Table 1:** Characteristics of the sample.

Population Attributes		N	(%)
Location	1. Village A	88	51.5
	2. Village B	83	48.5
Age (years)	1. 60–69	88	51.5
	2. 70–79	44	25.7
	3. ≥80	39	22.8
Gender	1. Male	99	57.9
	2. Female	72	42.1
Marital status	1. Single (Unmarried/Widowed)	35 (3/32)	20.5 (1.8/18.7)
	2. Married	136	79.5
Education	1. Illiteracy	72	42.1
	2. Literacy	99	57.9
Religious Belief	1. Illiteracy	63	36.8
	2. Literacy	108	63.1
Living arrangement	1. Alone	20	11.7
	2. Not alone	151	88.3
Personal competence			
ADL	1. Independent	108	63.2
	2. With disability	63	36.8
Mental state	1. Independent	103	60.2
	2. With disability	68	39.8
Perception Communication	1. Independent	119	69.6
	2. With disability	52	30.4
Social participation	1. Independent	134	78.4
	2. With disability	37	21.6
Subjective demands			
Daily care services	1. Not in need	100	58.5
	2. In need/Whatever	71	41.5
Medical care services	1. Not in need	62	36.3
	2. In need/Whatever	109	63.7
Educational services	1. Not in need	100	58.5
	2. In need/Whatever	71	41.5
Recreational services	1. Not in need	76	44.4
	2. In need/Whatever	95	55.6
Self-presenting services	1. Not in need	119	69.6
	2. In need/Whatever	52	30.4

ADL—activities of daily living.

**Table 2:** Relation between activities to personal attributes, competence and subjective demand.

		PW (N, %)					HW (N, %)					L (N, %)			
		0	<4	4–8	>8	X^2^	0	<4	4–8	>8	X^2^	<4	4–8	>8	X^2^
Location	A	46 (52.3)	9 (10.2)	11 (12.5)	22 (25)	4.441	47 (53.4)	22 (25)	11 (12.5)	8 (9.1)	3.063	28 (31.8)	25 (28.4)	35 (39.8)	1.234
	B	36 (43.4)	13 (15.7)	18 (21.7)	16 (19.3)		47 (56.6)	16 (19.3)	16 (19.3)	4 (4.8)		33 (39.8)	22 (26.5)	28 (33.7)	
Gender	Male	43 (43)	15 (15)	20 (20)	22 (22)	3.404	60 (60)	24 (24)	13 (13)	3 (3)	**8.177** *	34 (34)	25 (25)	41 (41)	1.860
	Female	39 (54.9)	7 (9.9)	9 (12.7)	16 (22.5)		34 (47.9)	14 (19.7)	14 (19.7)	9 (12.7)		27 (38)	22 (31)	22 (31)	
Age	60–69	34 (39.1)	12 (13.8)	20 (23)	21 (24.1)	10.415	52 (59.8)	15 (17.2)	14 (16.1)	6 (6.9)	**21.164** **	36 (41.4)	25 (28.7)	26 (29.9)	4.824
	70–79	26 (59.1)	7 (15.9)	2 (4.5)	9 (20.5)		21 (47.7)	6 (13.6)	12 (27.3)	5 (11.4)		15 (34.1)	11 (25)	18 (40.9)	
	≥80	22 (55)	3 (7.5)	7 (17.5)	8 (20)		21 (52.5)	17 (42.5)	1 (2.5)	1 (2.5)		10 (25)	11 (27.5)	19 (47.5)	
Education	Illiteracy	36 (50)	10 (13.9)	12 (16.7)	14 (19.4)	0.648	39 (54.2)	21 (29.2)	7 (9.7)	5 (6.9)	5.614	25 (34.7)	19 (26.4)	28 (38.9)	0.227
	Educated	46 (46.5)	12 (12.1)	17 (17.2)	24 (24.2)		55 (55.6)	17 (17.2)	20 (20.2)	7 (7.1)		36 (36.4)	28 (28.3)	35 (35.4)	
Religious Belief	Have	45 (41.7)	15 (13.9)	22 (20.4)	26 (24.1)	5.118	62 (57.4)	20 (18.5)	19 (17.6)	7 (6.5)	2.850	38 (35.2)	31 (28.7)	39 (36.1)	0.220
	Haven’t	37 (58.7)	7 (11.1)	7 (11.1)	12 (19)		32 (50.8)	18 (28.6)	8 (12.7)	5 (7.9)		23 (36.4)	16 (25.4)	24 (38.1)	
Marriage	Alone/Widow	19 (54.3)	5 (14.3)	4 (11.4)	7 (20)	1.328	20 (57.1)	9 (25.7)	5 (14.3)	1 (2.9)	1.428	9 (25.7)	9 (25.7)	17 (48.6)	2.917
	Married	63 (46.3)	17 (12.5)	25 (18.5)	31 (22.8)		74 (54.4)	29 (21.3)	22 (16.2)	11 (8.1)		52 (38.2)	38 (27.9)	46 (33.8)	
ADL	Independent	51 (47.2)	12 (11.1)	23 (21.3)	22 (20.4)	4.438	65 (60.2)	21 (19.4)	16 (14.8)	6 (5.6)	3.537	45 (41.7)	30 (27.8)	33 (30.6)	**6.106** *
	disability	31 (49.2)	10 (15.9)	6 (9.5)	16 (25.4)		29 (46)	17 (27)	11 (17.5)	6 (9.5)		16 (25.4)	17 (27)	30 (47.6)	
Mental state	Independent	47 (45.6)	15 (14.6)	19 (18.4)	22 (21.4)	1.296	56 (54.4)	20 (19.4)	20 (19.4)	7 (6.8)	3.111	38 (36.9)	32 (31.1)	33 (32)	2.940
	disability	35 (51.5)	7 (10.3)	10 (14.7)	16 (23.5)		38 (55.9)	18 (26.5)	7 (10.3)	5 (7.4)		23 (33.8)	15 (22.1)	30 (44.1)	
Perception communication	Independent	56 (47.1)	17 (14.3)	21 (17.6)	25 (21)	1.047	66 (55.5)	24 (20.2)	20 (16.8)	9 (7.6)	1.183	41 (34.5)	35 (29.4)	43 (36.1)	0.744
	disability	26 (50)	5 (9.6)	8 (15.4)	13 (25)		28 (53.8)	14 (26.9)	7 (13.5)	3 (5.8)		20 (38.5)	12 (23.1)	20 (38.5)	
Social participation	Independent	65 (48.5)	17 (12.7)	23 (17.2)	29 (21.6)	0.164	75 (56)	28 (20.9)	22 (16.4)	9 (6.7)	0.838	47 (35.1)	37 (27.6)	50 (37.3)	0.103
	disability	17 (45.9)	5 (13.5)	6 (16.2)	9 (24.3)		19 (51.4)	10 (27)	5 (13.5)	3 (8.1)		14 (37.8)	10 (27)	13 (35.1)	
Daily care service	In-need	50 (50)	13 (13)	11 (11)	26 (26)	6.804	50 (50)	26 (26)	16 (16)	8 (8)	2.967	36 (36)	31 (31)	33 (33)	2.055
	Not-in-need	32 (45.1)	9 (12.7)	18 (25.4)	12 (16.9)		44 (62)	12 (16.9)	11 (15.5)	4 (5.6)		25 (35.2)	16 (22.5)	30 (42.3)	
Health service	In-need	30 (48.4)	7 (11.3)	4 (6.5)	21 (33.9)	**12.463** **	34 (54.8)	13 (21)	11 (17.7)	4 (6.5)	0.348	24 (38.7)	18 (29)	20 (32.3)	0.891
	Not-in-need	52 (47.7)	15 (13.8)	25 (22.9)	17 (15.6)		60 (55)	25 (22.9)	16 (14.7)	8 (7.3)		37 (33.9)	29 (26.6)	43 (39.4)	
Educational service	In-need	50 (50)	12 (12)	16 (16)	22 (22)	0.487	50 (50)	24 (24)	18 (18)	8 (8)	2.502	37 (37)	30 (30)	33 (33)	1.638
	Not-in-need	32 (45.1)	10 (14.1)	13 (18.3)	16 (22.5)		44 (62)	14 (19.7)	9 (12.7)	4 (5.6)		24 (33.8)	17 (23.9)	30 (42.3)	
Recreational service	In-need	36 (47.4)	8 (10.5)	11 (14.5)	21 (27.6)	2.891	40 (52.6)	13 (17.1)	18 (23.7)	5 (6.6)	7.186	34 (44.7)	21 (27.6)	21 (27.6)	**6.302** *
	Not-in-need	46 (48.4)	14 (14.7)	18 (18.9)	17 (17.9)		54 (56.8)	25 (26.3)	9 (9.5)	7 (7.4)		27 (28.4)	26 (27.4)	42 (44.2)	
Self-presenting service	In-need	51 (42.9)	17 (14.3)	21 (17.6)	30 (25.2)	4.414	65 (54.6)	29 (24.4)	19 (16)	6 (5)	3.005	45 (37.8)	36 (30.3)	38 (31.9)	4.153
	Not-in-need	31 (59.6)	5 (9.6)	8 (15.4)	8 (15.4)		29 (55.8)	9 (17.3)	8 (15.4)	6 (11.5)		16 (30.8)	11 (21.2)	25 (48.1)	

** and * represent significance at 1% and 5%, respectively. Bold is used to highlight the items with significant results. PW—paid work; HW—housework; L—leisure activities; ADL—activities of daily living.
